# The New King's College Hospital, Denmark Hill

**Published:** 1911-03-04

**Authors:** 


### THE NEW KING'S COLLEGE HOSPITAL, DENMARK HILL.

On Saturday last the Building Committee of the new King's College Hospital invited a number of Press representatives to inspect the hospital buildings, or rather those blocks which are rapidly approaching completion on the slope of Denmark Hill. The fine site on which the new buildings stand was presented in 1904 by the Hon. W. Smith, Chairman of the Hospital, and after Mr. Pite's design, selected after competition, had been accepted, building operations were started with a nucleus of a special building fund obtained by appeal. In July 1909 his late Majesty King Edward VII. laid the foundation-stone of the central block, a ceremony to which a melancholy interest attaches, since it was the last action of the kind performed by his Majesty.

We hope on a future occasion to give an extended description of the hospital, with plans and detailed criticism. Last Saturday's hurried view of the premises already completed, or in course of completion, was scarcely sufficient, in justice to those who are concerned in this gigantic undertaking, to permit of such description or estimate. We must, therefore, content ourselves with a brief outline of what has so far been accomplished. The greater part of the administrative block and almost the whole of the out-patient block have been completed so far as the mere building, apart from fittings and installation, is concerned. In going through the latter one is at once struck with the fine proportions of the scheme, and the architectonic effect is substantiated by a glance at the beautiful entrance lobby of the administrative block which, when finished, should entitle this institution to rank easily first among London hospitals. The out-patient waiting-hall, too, is particularly attractive. It has a capacity of over 500, and the environing corridor that separates the examining-rooms from the central hall is a notable feature that will be much appreciated by staff and patients in the future. The day wards arranged above the out-patient department for casual cases offer another interesting feature of the scheme which is also heartily to be commended. The ventilation of the out-patient department, so far as can be judged at present, appears to be excellent. The dispensary waiting-hall and the throat and ear departments also offer unique features which need a more detailed description, while the excellent provision made for the almoners ought to be an example to all institutions that contemplate rebuilding.

The administrative block has been placed centrally, commanding all the various departments. A point that strikes the visitor at once is the fact that the nursing accommodation has been provided for in this block. Provision is made on two floors for nurses' rooms, lavatory accommodation, mess rooms, sisters' and matrons' rooms.

The material employed in building the hospital is for the most part ferro-concrete, with yellow brick, red dressings, and a moderate amount of Portland stone. The roofs are to be mainly flat and of asphalte, and are to be covered with green Westmorland slates. The lighting has been provided for by a separate installation, and the heating is to be by low-pressure steam. The question of floor material is still under consideration.

The pressmen had the benefit of expert guidance from the architect, Mr. W. A. Pite, F.R.I.B.A., the consulting engineer, Mr. D. S. Capper, M.I.C.E., and the clerk of works. At the conclusion of the inspection they were entertained to luncheon, and the Chairman of the Building Committee made a brief statement with regard to the needs of the hospital to which we allude in more detail in "Week's Work."

